# Supplementary material for: Examining community perspectives on integrated service delivery for tuberculosis, mental health and substance use disorder in Nigeria: A qualitative study
Source: PLoS One. 2026 Apr 3;21(4):e0345182. doi: 10.1371/journal.pone.0345182 (PMC13048408; doi:10.1371/journal.pone.0345182)
Supplement: Attachment — Submitted filename: Response_to_Reviewers_auresp_2.pdf [file pone.0345182.s004.pdf]

Response to Reviewers

Editor's comment

Introduction
1. The current version of the introduction is a significant improvement over the initial submission. Nevertheless, a critical gap in the review of pertinent literature highlighting what we already know about integrating TB, mental health and substance disorders, is missing in the introduction. I have included some published articles (this is by no means exhaustive) to highlight existing studies that the authors could use to fill this important gap in the introduction. The authors should situate this review immediately after the current paragraph 3.
I. Sweetland, A. C., Gruber Mann, C., Fernandes, M. J., Silva, F. V. S. de M., Matsuzaka, C., Cavalcanti, M., Fortes, S., Kritski, A., Su, A. Y., Ambrosio, J. C., Kann, B., & Wainberg, M. L. (2024). Barriers and Facilitators to Integrating Depression Treatment Within a TB Program and Primary Care in Brazil. Health Promotion Practice, 25(6), 1032–1039. https://doi.org/10.1177/15248399231183400
II. Todowede, O., Afaq, S., Adhikary, A., Kanan, S., Shree, V., Jennings, H. M., Faisal, M. R., Nisar, Z., Khan, I., Desai, G., Huque, R., & Siddiqi, N. (2023). Barriers and facilitators to integrating depression care in tuberculosis services in South Asia: a multi-country qualitative study. BMC Health Services Research, 23(1), Article 818. https://doi.org/10.1186/s12913-023-09783-z
III. Foo, C. D., Shrestha, P., Wang, L., Du, Q., García-Basteiro, A. L., Abdullah, A. S., & Legido-Quigley, H. (2022). Integrating tuberculosis and noncommunicable diseases care in low- and middle-income countries (LMICs): A systematic review. PLoS Medicine, 19(1), e1003899. https://doi.org/10.1371/journal.pmed.1003899
IV. Afaq, S., Ayub, A., Faisal, M. R., Nisar, Z., Zala, Rehman, A. ur, Ahmed, A., Todowede, O., & Siddiqi, N. (2024). Depression care integration in tuberculosis services: A feasibility assessment in Pakistan. Health Expectations : An International Journal of Public Participation in Health Care and Health Policy, 27(1), e13985-n/a. https://doi.org/10.1111/hex.13985
V. Heunis, C., & Kigozi-Male, G. (2024). Exploring Managers' Insights on Integrating Mental Health into Tuberculosis and HIV Care in the Free State Province, South Africa. International Journal of Environmental Research and Public Health, 21(11), 1528. https://doi.org/10.3390/ijerph21111528
VI. Lovero, K. L., Lammie, S. L., van Zyl, A., Paul, S. N., Ngwepe, P., Mootz, J. J., Carlson, C., Sweetland, A. C., Shelton, R. C., Wainberg, M. L., & Medina-Marino, A. (2019). Mixed-methods evaluation of mental healthcare integration into tuberculosis and maternal-child healthcare services of four South African districts. BMC Health Services Research, 19(1), Article 83. https://doi.org/10.1186/s12913-019-3912-9

Author response

Thanks for the observation and the referred manuscripts as indicated in the comment above. These are very relevant manuscripts when it comes to integration of services. However, a close scrutiny will reveal that the focus of the studies as listed differed so much from ours which elicited the views of beneficiaries of integrated service delivery and their

caregivers. It is important to point out that almost all the studies were referenced in the discussion section where they suited the focus of our manuscript.

Editor's comment
Methods
2. In lines 178 and 195, change interview to focus group discussion.

Author response
Corrections made. Thanks.

Editor's comment
3. In the data management sub-section, be explicit by stating that the study analyzed the data thematically before describing the process.

Author response
Included

Editor's comment
4. Move lines 225-229, "Interviewer characteristics" subsection to the "Study instrument and data collection method" subsection. Interviewer characteristics does not need to be a separate subsection.

Author response
This has been effected.

Editor's comment
Discussion
5. In line with the review of pertinent literature in the introduction, the authors should revise the discussion, by comparing the findings of the current study to results of existing scholarship.
Author response
They referred manuscripts were included in the discussion section.

Editor's comment
6. In line 512, replace "a number of participants" with "few participants".

Author response
Correction has been made.
Editor's comment
7. Lines 512-529 contains 3 key findings that should be discussed in separate paragraphs – safety

of mentally ill patients from contracting TB, a lack of medical specialization, and increased waiting time. The authors' argument that the comments on risk of mentally ill people contracting TB are contained in about four lines of the discussion, which may not be able to stand as a paragraph, is defective. Secondly, it is true that risk of contracting TB and lack of medical specialization 'are focused on the same concept.' The authors should be diligent enough to discuss the findings in separate paragraphs because they can stand own their own.

Author response

This has been done. Thanks.

Editor's comment

8. Move lines 543-544 to the preceding paragraph (lines 531-541) to ensure smooth transition to the succeeding paragraph.

Author response

Thanks for the observation. However, moving lines 543-544 to lines 531-541 will undermine the opening statement in discussing the third and most important theme of the study which may not be appropriate. Moreover, lines 543-544 is another opening statement that supports the statements in the preceding paragraph (lines 531-541) which is the opening statement of the third theme of the study.
