# Supplementary material for: Examining community perspectives on integrated service delivery for tuberculosis, mental health and substance use disorder in Nigeria: A qualitative study
Source: PLoS One. 2026 Apr 3;21(4):e0345182. doi: 10.1371/journal.pone.0345182 (PMC13048408; doi:10.1371/journal.pone.0345182)
Supplement: Attachment — Submitted filename: Reviewer Recommendation and Comments for Manuscript.docx [file pone.0345182.s001.docx]

**Recommendation**

The manuscript needs to be resubmitted with revisions

Overall evaluation

- Congratulations on the study highlighting the importance of integrated care for tuberculosis, mental health, and substance abuse, which is essential for effective tuberculosis control, especially in high-burden countries.

- Below are some comments to positively contribute to the enhancement of the study:

- Title: The title is appropriate, concise, and coherent with the study content. I recommend adding at the end of the title a colon followed by the specification of the study type, which will facilitate immediate identification of the research nature by readers.

- Abstract: The abstract is adequate, containing a clear synthesis aligned with the research objective.

- Introduction: The introduction covers relevant national and international concepts and data. However, the study would benefit from including a description of how tuberculosis care is provided in Nigeria, as well as access to health services in the country. In many countries, such care is offered in an integrated manner, especially driven by Primary Health Care, and international literature presents numerous promising examples that reinforce this integrated approach. It would be enriching to understand how Nigeria acts in disease control, considering that tuberculosis care still seems fragmented. Furthermore, given the study’s objective interrelating tuberculosis, mental health, and substance use as a chain perpetuating the disease, it would be pertinent to clarify how psychiatric services function in Nigeria, for example, whether they cover all types of mental disorders, including less severe cases, since it is mentioned that only hospitals fulfill this role. Although some methodological aspects briefly address this issue, we suggest that the introduction also include information about available mental health services and which substances are involved in substance abuse.

- Methodology: It is recommended to include the following information to enrich this section:

- In the section regarding data collection instruments, highlight that the instrument used was a semi-structured interview; specify which questions were used in the focus group discussions, even if based on prior pilot studies.

- In data management, inform how many researchers participated in the transcription, and whether any software was used for this step or if it was performed manually.

- Detail the technique used for qualitative data analysis. Although the use of QDA Miner Lite v2.0.6 software was mentioned, it is important to specify the analytical technique employed, whether thematic, discourse analysis, or other, and to detail it for replicability.

Results:

- I suggest removing the first paragraph of the results section and including this information in the methodology, adding that interviews were conducted by two researchers experienced in the topic; it is not necessary to name them.

- Regarding subject characterization, as the methodology mentions that 50% of participants were affected individuals and 50% were family members, it would be important to clarify the later percentages to avoid doubts about which groups they refer to. I recommend including a table detailing the sociodemographic profile of participants, appropriately segmented by group.

- About the selection of statements, it is important to mention in the methodology the analytical technique to understand how responses were selected, given that 12 focus groups with 116 individuals were used.

Discussion:

- It is recommended to incorporate international examples of successful integrated models to broaden the translation and applicability of the results. Additionally, a brief discussion of primary health care relating to the study findings would be relevant. From this perspective, adding examples of integrated approaches from countries with better disease control could further enrich the discussion.

Conclusion:

- Currently, the conclusion is somewhat generic. I recommend strengthening it based on the main findings of the study, highlighting lessons learned. It would also be important to include a section describing the research limitations, bringing transparency and foundation to the interpretations.
